# Supplementary material for: Neuroimaging Examination of Driving Mode Switching Corresponding to Changes in the Driving Environment
Source: Front Hum Neurosci. 2022 Feb 18;16:788729. doi: 10.3389/fnhum.2022.788729 (PMC8895376; doi:10.3389/fnhum.2022.788729)
Supplement: Supplementary file 1 [file Data_Sheet_1.docx]

Supplementary Information

**Neuroimaging examination of driving mode switching corresponding to changes in the driving environment**

Ryu Ohata, Kenji Ogawa, and Hiroshi Imamizu

Correspondence to H.I (imamizu@gmail.com) or R.O (ryu.oohata@gmail.com)

**Supplementary Figure**


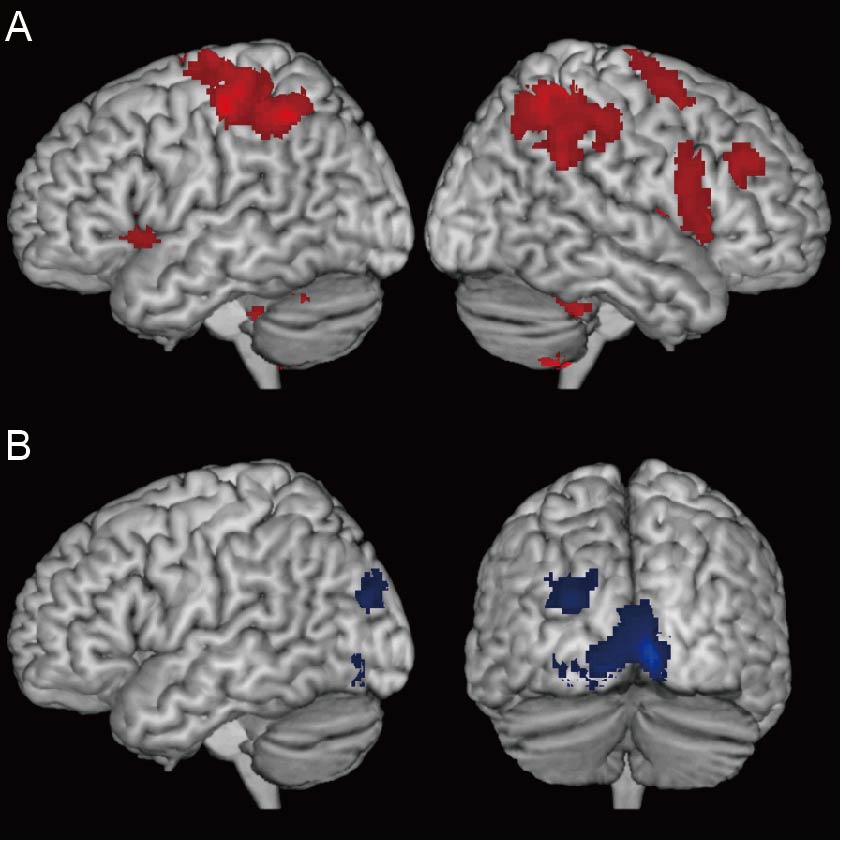


**Supplementary Figure 1**. Brain regions activated or deactivated in response to acceleration of preceding car. (A) Clusters of activation (in red) that significantly increased after the preceding car accelerated. (B) Clusters of activation (in blue) that significantly decreased after the preceding car accelerated. A threshold at *p* < 0.05 (FWE-corrected at cluster level with a cluster-forming threshold of *p* < 0.001) was set for statistical testing.


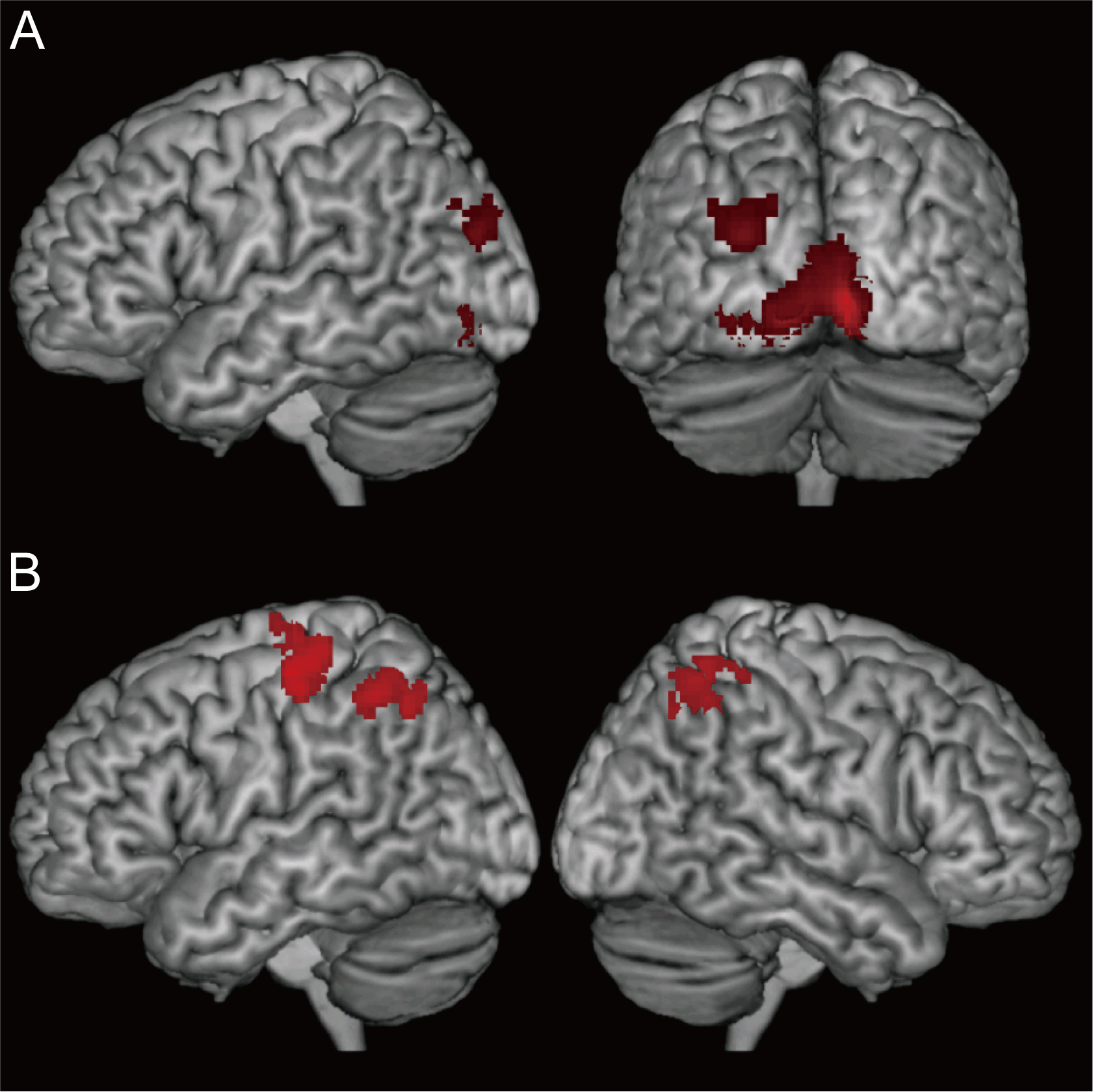


**Supplementary Figure 2**. Difference between activations involved in accelerator sensitivity change and those involved in preceding car’s acceleration. (A) Clusters of activation that were significantly larger in response to the changes in accelerator sensitivity than the preceding car’s acceleration. (B) Clusters of activation that were significantly larger in response to the preceding car’s acceleration than the changes in accelerator sensitivity. A threshold at *p* < 0.05 (FWE-corrected at cluster level with a cluster-forming threshold of *p* < 0.001) was set for statistical testing.

**Supplementary Movie S1**. Virtual car driving in a straight line, related to Figure 1B. The movie shows the car travel from the perspective of a driver (participant). The preceding blue car is seen in front of the participant’s car. The movie begins at the mid-point of the baseline period and ends at the end of the target period (see also Fig. 1B).
